# Increasing newly diagnosed inflammatory bowel disease and improving prognosis in China: a 30-year retrospective study from a single centre

**DOI:** 10.1186/s12876-020-01527-1

**Published:** 2020-11-12

**Authors:** Hong Lv, Meng Jin, Huimin Zhang, Xuanfu Chen, Meixu Wu, Mingyue Guo, Runing Zhou, Zheng Wang, Hong Yang, Jiaming Qian

**Affiliations:** Department of Gastroenterology, Peking Union Medical College Hospital, Peking Union Medical College, Chinese Academy of Medical Sciences, Beijing, 100730 China

**Keywords:** Inflammatory bowel disease, Prognosis, Epidemiology

## Abstract

**Background:**

We aimed to characterize the trends of prognosis in ulcerative colitis (UC) and Crohn’s disease (CD) in a Chinese tertiary hospital.

**Methods:**

A 30-year retrospective cohort analysis was conducted at Peking Union Medical College Hospital. Consecutive patients newly diagnosed with UC or CD from 1985 to 2014 were included. The primary outcome was in-hospital mortality. The secondary outcomes included surgery and length of stay in hospital. The Pearson correlation coefficient was applied to determine the relationship between time and prognosis. Multivariable logistic regression analysis was performed to determine the risk factors for in-hospital mortality and surgery.

**Results:**

In total, 1467 patients were included in this study (898 cases with UC and 569 cases with CD). Annual admissions for UC and CD have increased significantly over the last 30 years (UC, *r* = 0.918, *P* < 0.05; CD, *r* = 0.898, *P* < 0.05). Decreased in-hospital mortality was observed both in patients with UC and CD (UC, from 2.44 to 0.27%, *r* = − 0.827, *P* < 0.05; CD, from 12.50 to 0.00%, *r* = − 0.978, *P* < 0.05). A decreasing surgery rate was observed in patients with CD (*r* = − 0.847, *P* < 0.05), while an increasing surgery rate was observed in patients with UC (*r* = 0.956, *P* < 0.05). Shortened average lengths of hospital stay were observed in both UC and CD patients (UC, from 47.83 ± 34.35 to 23.58 ± 20.05 days, *r* = − 0.970, *P* < 0.05; CD, from 65.50 ± 50.57 to 26.41 ± 18.43 days, *r* = − 0.913, *P* < 0.05). Toxic megacolon and septic shock were independent risk factors for in-hospital mortality in patients with UC. Intestinal fistula and intestinal perforation were independent risk factors for in-hospital mortality in patients with CD.

**Conclusions:**

In this cohort, the admissions of patients with UC and CD were increased, with significantly improved prognoses during the past 30 years.

## Background

Inflammatory bowel disease (IBD) is a chronic idiopathic inflammation of the gastrointestinal tract. The inflammation of the intestinal mucosa in IBD is characterized by episodes of abdominal pain, diarrhea, bloody stools, weight loss, and the influx of neutrophils and macrophages that produce cytokines, proteolytic enzymes, and free radicals that result in inflammation and ulceration [1]. Currently, the prevalence of IBD surpasses 0.3% in Western society [2]. Moreover, over the last few decades, newly industrialized countries have also documented a rising incidence of IBD, which poses a heavy burden on global health systems due to high rates of productivity loss and severe complications [3, 4].

In China, the first case of IBD was reported in 1956. Since then, China has experienced a great acceleration in the incidence of IBD [5]. During the same time, treatment guidelines have evolved due to updating conceptions of disease pathogenesis [6]. However, how they affect surgical rates and long-term outcomes is still under investigation. Although several studies have reported short-term trends and hospitalization costs of IBD in China [4, 7–9], longitudinal trends of IBD in China still remain unclear.

Therefore, in order to better understand the longitudinal trends of IBD in China, we conducted this 30-year retrospective study to characterize the trends of admission, complication and prognosis in newly diagnosed IBD based on a hospital administrative database. We hope a better understanding of the longitudinal trends of IBD in China could offer valuable information for healthcare insurance management and assist in the discovery of novel therapeutic targets.

## Methods

### Patients

A 30-year retrospective, hospital-based study was undertaken at Peking Union Medical College Hospital. Diagnostic codes based on International Classification of Disease-9th Revision (ICD-9) and ICD-10 were used to search the hospital administrative database. Consecutive patients diagnosed with UC (ICD-9 556, ICD-10 K51) or CD (ICD-9 555, ICD-10 K50) from 1985 to 2014 were included. Records for all admissions were retrospectively reviewed by two gastroenterologists independently. Only patients with UC or CD as a primary diagnosis were included. For repeated admissions, only the first admission (with UC or CD as a primary diagnosis) was included. This study was approved by the Institutional Review Board of Peking Union Medical College Hospital.

### Data collection

Data concerning demographic characteristics, phenotypes, complications, surgeries and in-hospital mortality were extracted from the hospital administrative database. The primary outcome was in-hospital mortality. The secondary outcomes included the need for surgery, length of hospital stay and hospital costs.

### Statistical analysis

Continuous variables are presented as the mean ± SD and were analysed with Student’s *t*-test. Categorical variables were tested with the χ^2^ test or Fisher exact test. Trend analysis was analysed by Spearman correlation coefficient. Risk factors were identified by logistic regression analysis. All analyses were performed with SPSS 23 software (Chicago, IL, USA). *P* < 0.05 was considered statistically significant.

## Results

### Increasing newly diagnosed IBD

At first, 4114 cases were extracted from the administrative database. After data review, 1346 cases were excluded because IBD was not the primary discharge diagnosis, and 1301 cases were excluded because of repeated admissions.

At last, a total of 1467 cases were included in the final analysis, including 898 cases of UC (61.21%) and 569 cases of CD (38.79%). As shown in Table 1, the male ratio of patients with UC was lower than that of patients with CD (1.11:1 vs 1.96:1, *P* < 0.001). The average age at admission was younger in patients with UC compared with patients with CD (41.95 ± 15.36 years vs 37.62 ± 15.04 years, *P* < 0.001). The peak age at admission in patients with UC and CD was 30–39 years old and 20–29 years old, respectively.

**Table 1:** Characteristics of patients with IBD

	IBD	UC	CD	*P*
	*N* = 1467	*N* = 898	*N* = 569	
Sex, n (%)				
Male	849 (57.87)	472 (52.56)	377 (66.26)	< 0.001
Female	618 (42.13)	426 (47.44)	192 (33.74)	
Age (years)	40.27 ± 15.38	41.95 ± 15.36	37.62 ± 15.04	< 0.001
0–9	8 (0.55)	7 (0.78)	1 (0.18)	
10–19	98 (6.68)	43 (4.79)	55 (9.67)	
20–29	338 (23.04)	180 (20.04)	158 (27.77)	
30–39	321 (21.88)	201 (22.38)	120 (21.09)	
40–49	285 (19.43)	178 (19.82)	107 (18.80)	
50–59	225 (15.34)	152 (16.93)	73 (12.83)	
60–69	135 (9.20)	97 (10.80)	38 (6.68)	
70–79	51 (3.48)	37 (4.12)	14 (2.46)	
> 80	6 (0.41)	3 (0.33)	3 (0.53)	

The numbers of newly diagnosed patients with UC and CD increased significantly during the last 30 years. The newly diagnosed cases of UC increased from 41 cases in 1986–1990 to 373 cases in 2011–2015 (*r* = 0.918, *P* < 0.05). The newly diagnosed cases of CD increased from 16 cases in 1986–1990 to 276 cases in 2011–2015 (*r* = 0.898, *P* < 0.05).

### Improving prognosis of newly diagnosed IBD

A total of 20 patients died in this study, including 9 cases of UC (1.00%) and 11 case of CD (1.93%). The reasons for death included septic shock (7 cases, 35%), haemorrhagic shock (5 cases, 25%), multiple organ failure syndrome (4 cases, 20%), toxic megacolon (2 cases, 10%) and intestinal perforation (2 cases, 10%). The in-hospital mortality for patients with UC decreased from 2.4% in 1986–1990 to 0.3% in 2011–2015 (*r* = − 0.827, *P* < 0.05). The in-hospital mortality for patients with CD decreased from 12.5% in 1986–1990 to 0% in 2011–2015 (*r* = − 0.978, *P* < 0.05).

A total of 277 cases received operations, including 106 cases of UC (11.8%) and 171 cases of CD (30.1%). An increasing trend in the annual operation rate was observed in patients with UC (*r* = 0.956, *P* < 0.05), while a decreasing trend in the annual operation rate was observed patients with CD (*r* = − 0.847, *P* < 0.05).

Shortened average lengths of hospital stay were observed both in patients with UC and CD (UC, from 47.83 ± 34.35 to 23.58 ± 20.05 days, *r* = − 0.970, *P* < 0.05; CD, from 65.50 ± 50.57 to 26.41 ± 18.43 days, *r* = − 0.913, *P* < 0.05).

### Complications of IBD

As shown in Table 2, toxic megacolon (16 cases, 1.78%) was the most common severe complication of UC, followed by intestinal perforation (13 cases, 1.45%), intestinal obstruction (9 case, 1.00%), intestinal fistula (5 cases, 0.56%) and haemorrhagic shock (3 cases, 0.33%). Intestinal obstruction was the most common severe complication of CD (128 cases, 22.5%), followed by intestinal fistula (85 cases, 14.94%), intestinal perforation (27 cases, 4.75%) and haemorrhagic shock (5 cases, 0.88%). The annual proportion of complications varied from 34.62 to 39.68% during the study period in newly diagnosed patients with CD and varied from 3.70 to 7.32% in newly diagnosed patients with UC. No significant trend was observed in the proportion of complications in UC and CD.

**Table 2:** Complications in patients with UC and CD

	All	UC	CD	*P*
	*N* = 2315	*N* = 1313	*N* = 1002	
Intestinal obstruction, n (%)	137 (9.34)	9 (1.00)	128 (22.50)	< 0.001
Intestinal perforation, n (%)	40 (2.73)	13 (1.45)	27 (4.75)	< 0.001
Intestinal fistula, n (%)	90 (6.13)	5 (0.56)	85 (14.94)	< 0.001
Toxic megacolon, n (%)	16 (1.09)	16 (1.78)	0 (0.00)	< 0.001
Haemorrhagic shock, n (%)	8 (0.55)	3 (0.33)	5 (0.88)	0.168

### Risk factors for prognosis in IBD

As shown in Table 3, multivariable regression analysis showed that year of admission, toxic megacolon, septic shock and haematological comorbidities were independent risk factors for death in patients with UC. Intestinal fistula, intestinal perforation and septic shock were independent risk factors for surgery in patients with CD.

**Table 3:** Multivariable regression analysis of risk factors for in-hospitality death in IBD

Characteristics	β	SE	Wald	*P*
*UC*				
Year of admission	− 1.111	0.3	13.671	< 0.001
Age at admission	0.075	0.032	5.474	< 0.05
Toxic megacolon	7.393	2.907	6.47	< 0.05
Septic shock	7.188	2.673	7.232	< 0.05
Haematological comorbidities	6.137	1.734	12.524	< 0.001
Constant	− 5.291	2.696	3.851	0.05
*CD*				
Year of admission	− 0.940	0.310	9.217	0.002
Intestinal fistula	2.454	1.128	4.736	0.030
Intestinal perforation	3.460	1.325	6.819	0.009

β short for β regression coefficient; SE short for standard error; Wald short for Wald χ^2^ test

As shown in Table 4, intestinal obstruction, intestinal perforation, canceration, toxic megacolon and haemorrhagic shock were independent risk factors for surgery in patients with UC. Intestinal fistula, intestinal obstruction and intestinal perforation were independent risk factors for surgery in patients with CD.

**Table 4:** Multivariable regression analysis of risk factors for operation in IBD

Characteristics	β	SE	Wald	*P*
*UC*				
Age	0.018	0.007	7.239	< 0.05
Intestinal obstruction	2.304	0.527	19.09	< 0.001
Intestinal Perforation	4.001	0.835	22.947	< 0.001
Toxic megacolon	2.317	1.031	5.054	< 0.05
Haemorrhagic shock	2.707	1.013	7.141	< 0.05
Canceration	3.735	1.107	11.393	< 0.05
Septic shock	2.835	0.956	8.793	< 0.05
Constant	− 3.586	0.635	31.915	< 0.001
*CD*				
Year of admission	− 0.270	0.078	11.894	0.001
Intestinal fistula	0.765	0.291	6.923	0.009
Intestinal obstruction	1.114	0.238	21.947	0.000
Intestinal perforation	1.615	0.625	6.680	0.010

β short for β regression coefficient; SE short for standard error; Wald short for Wald χ^2^ test

## Discussion

Hospitalization, surgery and in-hospital mortality are important measurements for IBD management. A few studies have evaluated how these measurements have changed since the introduction of new conceptions of pathogenesis and treatment strategies [6, 7]. However, longitudinal trends in newly industrialized countries remain unclear. This longitudinal, hospital-based study points to an increase in annual admissions from 1985 to 2014. More importantly, our study revealed decreasing in-hospital mortality and shortened length of hospital stay in patients with IBD over the last 30 years, which mostly attribute to improved understanding and updated management of IBD.

At the turn of the twenty-first century, IBD became a global disease with accelerating incidence in the newly industrialized countries [2]. An estimated 2.5–3 million people in Europe are affected by IBD, with a direct healthcare cost of 4.6–5.6 billion Euros per year [3]. However, while the incidence and hospitalizations for IBD in Western countries remains stable or slowly declining, literature has shown an explosive trend in developing countries in the latest decade [3, 9]. Overall, the incidence of IBD in China is 3.3 per 100,000 [4, 5]. Our study showed that the annual admissions for UC and CD rose significantly over the last 30 years (UC, *r* = 0.918, *P* < 0.05; CD, *r* = 0.898, *P* < 0.05), which provides relatively longer information on trends.

More importantly, our study showed an improving prognosis for IBD during the study period after adjustment for complications and severe comorbidity. Decreasing in-hospital mortality and shortened length of hospital stay were observed in this cohort. Many factors may contribute to the improved prognosis of IBD, such as increased awareness of early detection and intervention, improved access to healthcare providers, improved surveillance system, and evolved treatment strategies. Image techniques including magnetic resonance enterography, virtual computed tomography colonoscopy have been upgraded to better facilitated IBD clinical management [10–12]. At the start of our study period, treatment options and strategies were different from now. Since the formal Chinese consensus of IBD was released in 2000, clinician’s awareness has been well improved, which may contribute to the decreasing in-hospital mortality.

Surgery also plays a vital role in the treatment of IBD. Surgery for IBD is mainly used to eliminate complications or when medical therapy fails [13]. A total of 277 cases received operations in this study, including 106 cases of UC (11.8%) and 171 cases of CD (30.1%), which was in accordance with the literature [14]. Changes in the surgery rate for IBD are controversial. A meta-analysis reported a decreasing surgery rate for CD over the past several decades [15], while some hospital-based studies reported conflicting results [16]. Our study showed a decreasing surgery rate in patients with CD (*r* = − 0.847, *P* < 0.05) and an increasing surgery rate in patients with UC (*r* = 0.956, *P* < 0.05). The changing pattern of surgery in patients with CD was in accordance with Ma et al., who reported a decreasing rate of surgery for CD by 3.5% per year (95% CI: − 1.1%, − 5.8%) from 2002 to 2010 in Canada [17]. The decrease in surgical rates might be due to evolving practice patterns, such as earlier disease detection and intervention, closer endoscopic follow-up and increasing adoption of immunosuppressive agents and biologic agents [18].

As for complications in IBD, our result showed that toxic megacolon (16 cases, 1.78%) was the most common severe complication in UC, while intestinal obstruction was the most common severe complication in CD (128 cases, 22.5%), followed by intestinal fistula (85 cases, 14.94%). Moreover, the above complications were independent risk factors for in-hospital mortality and surgery. However, no decrease in the occurrence of complications was observed in this study in the era of biologics. This might be explained by increasing awareness, early detection and close monitoring in the management of severe complications in IBD. Prospective cohort studies with more detailed adjustments in medical therapy are needed to confirm our results.

The present study has several limitations. The first is confounding bias. Due to the retrospective and hospital-based design of study, our results should be interpreted carefully. Our hospital is a tertiary single center, the severity of patients included in this study is higher than general IBD patients. Population-based study with better representative subjects is needed to confirm these results. Second, this study covered a span of 30 years. Over this time, there were changes in diagnostic criteria and management, technology and economy. However, regression analysis showed that year of admission was an independent risk factor for death, which means that the prognosis of UC has indeed improved during the last 30 years. Last, our study focused on short outcomes of newly diagnosed IBD patients, and further studies with longer follow-up information are needed to focus on the long-term outcomes of IBD patients. Therefore, this research only provides an overview of UC from a single centre and wishes to provide information for further research.

## Conclusion

This longitudinal, hospital-based study showed an increasing tendency in newly diagnosed IBD and, more importantly, an improving prognosis of patients with IBD. In addition, our results showed that severe complications also have an important effect on prognosis, and early recognition and intervention are crucial in further management.

## Data Availability

The datasets used and/or analysed during the current study are available from the corresponding author on reasonable request.

## Abbreviations

UC: Ulcerative colitis
CD: Crohn’s disease
IBD: Inflammatory bowel disease
