# Correction to: Increasing newly diagnosed inflammatory bowel disease and improving prognosis in China: a 30-year retrospective study from a single centre

**DOI:** 10.1186/s12876-021-02077-w

**Published:** 2022-03-08

**Authors:** Hong Lv, Meng Jin, Huimin Zhang, Xuanfu Chen, Meixu Wu, Mingyue Guo, Runing Zhou, Zheng Wang, Hong Yang, Jiaming Qian

**Affiliations:** Department of Gastroenterology, Peking Union Medical College Hospital, Peking Union Medical College, Chinese Academy of Medical Sciences, Beijing, 100730 China

## Correction to: BMC Gastroenterol (2020) 20:377 https://doi.org/10.1186/s12876-020-01527-1

After publication of this article [1], the authors reported that in the Results section, in the sentence “The in-hospital mortality for patients with CD decreased from 12.5% in 1986–1990 to 1.8% in 2011–2015”, the mortality in 2011–2015 was actually 0% rather than 1.8%.

The original article [1] has been updated.
